# The effect of preoperative smoking cessation and smoking dose on postoperative complications following radical gastrectomy for gastric cancer: a retrospective study of 2469 patients

**DOI:** 10.1186/s12957-019-1607-7

**Published:** 2019-04-02

**Authors:** Hu Quan, Linda Ouyang, Huijun Zhou, Yongzhong Ouyang, Hua Xiao

**Affiliations:** 1 Department of Gastroduodenal and Pancreatic Surgery, Hunan Cancer Hospital and the Affiliated Cancer Hospital of Xiangya School of Medicine, Central South University, No. 283 Tongzipo Road, Changsha, 410013 China; 2 Central Laboratory of Hunan Cancer Hospital and the Affiliated Cancer Hospital of Xiangya School of Medicine, Central South University, No. 283 Tongzipo Road, Changsha, 410013 China; 3 Department of Gastroenterology and Urology, Hunan Cancer Hospital and the Affiliated Cancer Hospital of Xiangya School of Medicine, Central South University, No. 283 Tongzipo Road, Changsha, 410013 China

**Keywords:** Gastric cancer, Gastrectomy, Postoperative complication, Cigarette smoking, Smoking cessation

## Abstract

**Background:**

To investigate whether smoking adversely affects the short-term outcomes and the potential effects of cigarette dose and preoperative smoking cessation, in patients who underwent gastric cancer (GC) surgery.

**Methods:**

Two thousand, four hundred sixty-nine consecutive patients who underwent radical gastrectomy from November 2010 to July 2018 were included in the present study. Smokers (current or former smokers) were divided into 3 groups in accordance with the duration of smoking cessation preoperatively (≤ 2, 2 to 4, or ≥ 4 weeks) and the cigarette dose (≤ 20, 20 to 40, and ≥ 40 pack-years). The primary endpoint was postoperative complications (surgical site infection, pulmonary problems, bleeding, and others).

**Results:**

A total of 1056 patients (42.8%) were smokers. Compared with non-smokers, smokers had significantly higher overall postoperative complications (11.3% vs 7.5%, *P* = 0.001), and in particular pulmonary problems. Smokers also had more major complications, needing intensive care unit care, and longer postoperative hospital stays. Multivariate analysis confirmed that smoking (odds ratio = 1.506, 95% confidence interval 1.131–2.004, *P* = 0.005) was an independent risk factor for postoperative complications. Further subgroup analysis identified that there was a positive relationship between the incidence of complications and cigarette dose, and > 20 pack-years was demonstrated to have increased significantly the risk of complications. Smokers who stopped smoking ≥ 4 weeks before surgery had lower pulmonary problems than those with a shorter period of smoking cessation.

**Conclusions:**

Preoperative smoking cessation should be encouraged to reduce postoperative complications in GC patients, especially for heavy smokers.

## Background

Gastric cancer (GC) is ranked as one of the most prevalent malignancies worldwide with about one half of cases occurring in China. At present, curative resection is the only mainstay of therapy [1, 2]. Despite a downward trend in the incidence of postoperative complications following gastrectomy, due to significant improvements in surgical techniques, about 10.3–23.6% of patients experienced morbidity, resulting in longer hospital stays, increased healthcare costs, and even perioperative death [3–6]. In order to reduce postoperative complications and improve the patients’ quality of life, it is essential to identify potential risk factors and those patients at high risk. To date, various factors have been identified as adverse predictors for morbidity after gastrectomy, such as advanced age, obesity, and blood transfusion [3, 5, 6]. Unfortunately, most of these factors are not modifiable [7]. There is growing evidence that smoking is a significant risk factor for postoperative complications following a variety of operations, especially pulmonary problems and surgical site infections [8–11]. As a result, quitting smoking before surgery is likely to improve the short-term outcomes [12]. Other scholars have argued that cessation of smoking shortly before surgery will not decrease postoperative complications [13, 14]. Possible explanations for the conflicting data were the variable duration of smoking cessation before surgery and inconsistency in patient inclusion criteria.

In fact, most of the previous studies were conducted in patients who underwent thoracic surgery or taking varied abdominal surgery procedures together. But in fact, the incidence and risk factors for postoperative pulmonary problems following lower and upper abdominal surgery may differ significantly. For example, patients who underwent hepatobiliary and upper gastrointestinal surgery were twice as likely to develop pulmonary complications than those from any other surgical categories [15]. Thus, to reduce postoperative complications, it is important to understand the incidence and risk factors for complications for each type of surgical procedure. To our knowledge, no study has focused solely on GC patients and the influence of cigarette dose on postoperative complications following gastrectomy has not been investigated [16]. We hypothesize that smoking may serve as an independent risk factor for postoperative complications following radical gastrectomy for GC, especially pulmonary problems, and there may be a dose-dependent effect between cigarette dose and the incidence of complications, and an optimal duration of smoking cessation preoperatively could reduce its incidence. This question was addressed by conducting a retrospective study to investigate the potential influence of smoking, cigarette dose, and the duration of smoking cessation prior to surgery on the incidence of postoperative complications in GC patients who underwent radical gastrectomy.

## Methods

### Design and patients

**Fig. 1:**
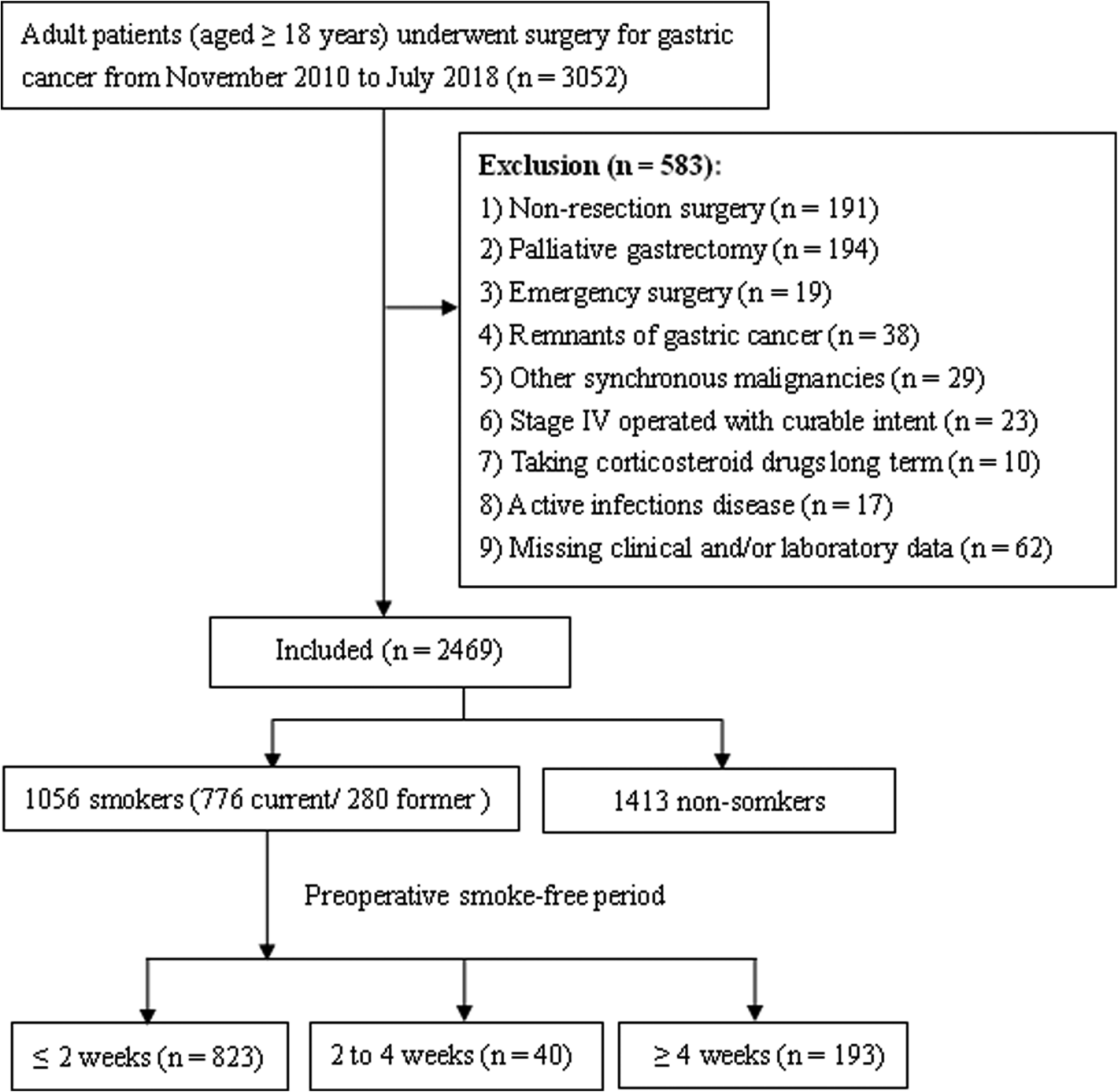
Flow chart

We retrospectively reviewed the medical record of all adult patients with GC who underwent operation in our hospital from November 2010 to July 2018. Exclusion criteria are described in Fig. 1. A total of 2469 consecutive patients were included in the present study. The smoking history of each patient was established by the responsible doctor on admission. Patients were divided into two groups, namely smokers and non-smokers according to their answers. For smokers, the average pack of cigarette smoked everyday and the number of years of smoking were recorded. All smokers were instructed to quit smoking immediately when they were admitted. Active and passive smoking was strictly prohibited for all patients during their hospital stay. The present study was approved by the ethics committee of our hospital, and informed consent was waived considering it is a retrospective investigation and observational in nature. The clinicopathological factors including baseline demographics, preoperative laboratory measurements, intraoperative variables, and pathological tumor characteristics (based on AJCC 8th edition [17]) were evaluated.

### Surgical procedures and perioperative management

Gastrointestinal surgeons with sufficient experience of curative radical gastrectomy performed or supervised all operations. Lymphadenectomy and digestive tract reconstruction were performed in accordance with the Japanese gastric cancer treatment guidelines [18]. As described in our previous studies [6, 19], for patients with locally advanced GC, open procedure with D2 or D2+ (no. 8p, 12p, 13, and/or 14v lymph nodes) lymphadenectomy was the main surgical type and all of the procedure was performed by a midline incision. But no patients were performed with D3 lymphadenectomy in the present study. While for those with early GC, total laparoscopy or laparoscopy-assisted gastrectomy with D1 + α or β lymphadenectomy was performed. Combined multi-organ resection was carried out in patients with a locally advanced tumor with potential invasion of nearby organs to achieve R0 resection, or resection of other organs due to simultaneously occurring benign disease. A second- or third-generation cephalosporin was used as a prophylactic antibiotic in all patients and administered for 3 to 5 days after surgery. The nasogastric tube was usually removed on postoperative day 1, but was left in place for about 3 days until anal exhaust occurred in some patients with severe edema of the stomach because of pyloric obstruction.

## Definitions

Smokers were defined as current or former smokers, and non-smokers were considered to be those who had never previously smoked. Smokers were divided into three categories depending on their smoking cessation period before surgery (≤ 2, 2 to 4, and ≥ 4 weeks), as reported for the majority of previous similar studies [12, 15, 20–22]. The cigarette dose was calculated by the average number of packs of cigarettes smoked everyday multiplied by the duration of smoking years (pack-years). In addition, smokers were subsequently classified into 3 subgroups according to the 25th and 75th cigarette dose, indicating light, moderate, and heavy smokers (20 and 40 pack-years in the present study, respectively), similar to but not exactly the same with a previous study, in which pack-year was divided into quartiles [9].

Postoperative complications were noted for ≤ 30 days following surgery and classified according to the Clavien-Dindo classification [23]. As reported by Aoyama et al. [24], grade I complications were excluded as they are considered to have little clinical significance. Major complications were defined as Clavien-Dindo grade III or greater. In some patients who suffered from more than one complication, each of the complications was recorded and analyzed.

## Statistical analysis

All patients were classified into two groups according to whether they had ever smoked or not. Differences between the groups were compared by univariate analyses, a *χ*2 or Fisher’s exact test for categorical variables. The test data for normal distribution was checked by the Shapiro-Wilk test, and a Student *t* test or Mann-Whitney *U* test was used for continuous data which was normally distributed or not. Continuous and multi-classification categorical variables were converted into binary outcomes according to the standard clinical or widely accepted thresholds, as reported in our previous studies [6, 19]. Univariate analysis by a *χ*^2^ test was carried out to test the impact of clinicopathological variables on postoperative complications. Multivariate binary logistic regression analysis (forward: LR) was further used to test factors with a *P* value < 0.05 following univariate analysis. The prognostic performance of the model was evaluated by the area under the curve (AUC) for the receiver operator characteristic (ROC) plot. Subgroup analysis was performed basing on the smoking cessation period before surgery and the pack-years and was compared with the non-smoker group as the reference using logistic regression analysis. All statistical analyses were performed with IBM SPSS Statistics for Windows (Version 24, NY: IBM Corporation, US), and a *P* value < 0.05 was considered to be significant.

## Results

### Characteristics of patients

Baseline demographics of the entire cohort of 2469 patients are described in Table 1. The mean age was 55.44 years (range 19–86), mean body mass index (BMI) was 21.83 kg/m^2^ (range 13.84–35.17), and 1625 (65.8%) of the patients were male. The majority (74.0%) underwent distal subtotal gastrectomy, and the remainder underwent either total (23.5%) or proximal subtotal (2.5%) gastrectomy. Based on the 8th edition of the TNM classification, there were 648 (26.2%) stage I, 544 (22.0%) stage II, and 1277 (51.7%) stage III patients. The mean duration of surgery was 199.28 min (range 70–584), and the mean estimated intraoperative bleeding was 203.41 mL (rang, 30–2300).

**Table 1:** Clinicopathological characteristics of the entire cohort (*n* = 2469)

Variables	Smokers (*n* = 1056)	Non-smokers (*n* = 1413)	*P* value
Gender (male)	1042 (98.7%)	582 (41.1%)	< 0.001
Age (years)	57.02 ± 9.37	54.26 ± 11.28	< 0.001
Body mass index (kg/m^2^)	21.87 ± 3.00	21.80 ± 2.98	0.574
ASA score			< 0.001
1	151 (14.3%)	288 (20.4%)	
2	789 (74.7%)	975 (69.0%)	
3	109 (10.3%)	147 (10.4%)	
4	7 (0.7%)	3 (0.2%)	
Any comorbidities	353 (33.4%)	384 (27.2%)	0.001
Chronic obstructive pulmonary disease	54 (5.1%)	27 (1.9%)	< 0.001
Abdominal surgery history	82 (7.8%)	180 (12.7%)	< 0.001
Pulmonary function test^a^			
Predicted vital capacity (%VC)	87.36 ± 19.45	89.95 ± 20.67	0.004
Forced expiratory volume in 1 s (FEV1%)	87.88 ± 12.49	90.18 ± 10.34	< 0.001
Preoperative albumin (g/L)	38.14 ± 4.59	38.49 ± 4.51	0.056
Preoperative hemoglobin (g/L)	123.62 ± 25.56	115.38 ± 23.34	< 0.001
Complication due to the tumor^b^	236 (22.3%)	298 (21.1%)	0.452
Operation method			0.539
Open	808 (76.5%)	1096 (77.6%)	
Laparoscopy	248 (23.5%)	317 (22.4%)	
Type of resection			0.914
Proximal subtotal gastrectomy	25 (2.4%)	36 (2.5%)	
Distal subtotal gastrectomy	779 (73.8%)	1048 (74.2%)	
Total gastrectomy	252 (23.9%)	329 (23.3%)	
Extent of lymph node dissection			0.364
≥ D2	998 (94.5%)	1323 (93.6%)	
< D2	58 (5.5%)	90 (6.4%)	
Combined multi-organ resection	98 (9.3%)	122 (8.6%)	0.577
Tumor size (cm)	4.15 ± 2.02	4.03 ± 2.12	0.162
Tumor location			< 0.001
Upper	113 (10.7%)	103 (7.3%)	
Middle	211 (20.0%)	302 (21.4%)	
Lower	713 (67.5%)	945 (66.9%)	
Diffuse	19 (1.8%)	63 (4.5%)	
T stage^c^			0.032
T1	196 (18.6%)	297 (21.0%)	
T2	167 (15.8%)	191 (13.5%)	
T3	82 (7.8%)	79 (5.6%)	
T4	611 (57.9%)	846 (59.9%)	
N stage^c^			0.490
N0	402 (38.1%)	567 (40.1%)	
N1	184 (17.4%)	225 (15.9%)	
N2	208 (19.7%)	257 (18.2%)	
N3	262 (24.8%)	364 (25.8%)	
pTNM stage^c^			0.890
I	272 (25.8%)	376 (26.6%)	
II	235 (22.2%)	309 (21.9%)	
III	549 (52.0%)	728 (51.5%)	
Harvested lymph node	22.70 ± 8.95	21.30 ± 8.38	< 0.001
Intraoperative blood loss (mL)	208.51 ± 126.73	199.60 ± 118.58	0.073
Operation time (min)	203.97 ± 54.61	195.77 ± 53.45	< 0.001
Postoperative complications^d^	119 (11.3%)	106 (7.5%)	0.001
Transferring to ICU postoperation	33 (3.1%)	25 (1.8%)	0.028
Postoperative hospital stays (days)	11.78 ± 6.63	11.17 ± 4.46	0.007

Data are presented as mean ± SD or n (%)

*ASA* American Society of Anesthesiology, *ICU* intensive care unit

^a^Data for 395 patients were missing (130 smokers, 265 non-smokers)

^b^Including pyloric obstruction or bleeding

^c^Tumor stages are based on the 8th edition of AJCC TNM classification

^d^Data are no. of patients (%), patients may have more than one complication

One thousand and fifty-six (42.8%) patients were smokers, with a median cigarette dose of 30 pack-years (range 1–120), and the remaining 1413 cases were non-smokers. As shown in Table 1, smokers were significantly male predominant and of advanced age, with comorbidities (including chronic obstructive pulmonary disease, COPD), worse pulmonary function (smaller predicted vital capacity (%VC), forced expiratory volume in 1 s (FEV1%)), upper third location of the tumor, more harvested lymph nodes, longer operation times, adverse postoperative events, and a higher frequency of requiring intensive care (all *P* < 0.05). The mean duration of the postoperative hospital stay was also longer than for the non-smoker group (11.78 vs 11.17 days, *P* = 0.007). It is noteworthy that the type of resection, operation method, pathological TNM stages, and mean BMI and tumor size were comparable between the two groups (all *P* > 0.05).

### Postoperative complications

Two hundred and ninety-three postoperative adverse events occurred in 220 patients of the entire cohort (8.9%), with surgical site infections being the most common (*n* = 116), followed by pulmonary complications (*n* = 100), bleeding (*n* = 24), and others (*n* = 53) (see Table 2). Among surgical site infections, organ/space surgical site infection occurred most frequently, including 63 cases of intra-abdominal infections, 26 cases of anastomotic or stump leakage, and so on. With respect to pulmonary problems, pneumonia was the most common, which occurred in 65 patients, with 63 were bacterial and the remaining 2 cases were aspiration. Based on the Clavien-Dindo classification, the incidence of stage II, IIIa, IIIb, IVa, IVb, and V complications was 8.3% (*n* = 206), 1.3% (*n* = 32), 1.3% (*n* = 31), 0.6% (*n* = 15), 0.04% (*n* = 1), and 0.3% (*n* = 8), respectively. Eighty-nine events of major complications (≥ grade III) occurred in 69 patients.

**Table 2:** Postoperative complications and severity of complications in smokers and non-smokers (293 events in 220 patients)

Variables	Smokers (*n* = 1056)	Non-smokers (*n* = 1413)	*P* value
Complications^a^	150 (14.2%)	143 (10.1%)	0.002
Surgical site infection	57 (5.4%)	59 (4.2%)	0.156
Organ/space surgical site infection	53 (5.0%)	51 (3.6%)	
Would dehiscence	4 (0.4%)	8 (0.6%)	
Pulmonary complication	54 (5.1%)	46 (3.3%)	0.020
Pneumonia	32 (3.0%)	33 (2.3%)	
Pleural effusion	15 (1.4%)	10 (0.7%)	
Acute respiratory distress syndrome	2 (0.2%)	0	
Pneumothorax	3 (0.3%)	0	
Atelectasis	2 (0.2%)	3 (0.2%)	
Bleeding	12 (1.1%)	12 (0.8%)	0.472
Intra-abdominal bleeding	6 (0.6%)	9 (0.6%)	
Gastrointestinal bleeding	6 (0.6%)	3 (0.2%)	
Others	27 (2.6%)	26 (1.8%)	0.224
Intestinal obstruction	12 (1.1%)	8 (0.6%)	
Ascites	9 (0.5%)	6 (0.4%)	
Cerebral infarction	2 (0.2%)	2 (0.1%)	
Delayed gastric emptying	1 (0.1%)	1 (0.1%)	
Anastomotic stricture	0	2 (0.1%)	
Liver failure	1 (0.1%)	1 (0.1%)	
Urinary tract infection	1 (0.1%)	1 (0.1%)	
Cardiac arrest	1 (0.1%)	1 (0.1%)	
Urinary retention	0	2 (0.1%)	
Diabetic ketoacidosis	0	1 (0.1%)	
Renal failure	0	1 (0.1%)	
Severity of complications^b^			0.005
Minor (grade II)	102 (9.8%)	104 (7.4%)	
Major (grade III or greater)	48 (4.5%)	39 (2.8%)	
Grade IIIa	17 (1.6%)	15 (1.1%)	
Grade IIIb	18 (1.7%)	13 (0.9%)	
Grade IVa	8 (0.8%)	7 (0.5%)	
Grade IVb	0	1 (0.1%)	
Grade V	5 (0.5%)	3 (0.2%)	

^a^Data are number of complications (%), patients may have more than one complications

^b^Based on the Clavien-Dindo severity classification of surgical complications

When comparing the overall incidence of postoperative complications stratified by smoking history, the rate was significantly greater in smokers than in non-smokers (11.3% vs 7.5%, *P* = 0.001). Considering each type of complication, only pulmonary problems were significantly more common in the smoker group. However, the incidence of surgical site infection, bleeding, and other complications was comparable between the two groups. Forty-eight cases of major complications occurred in 39 smokers, which was substantially more frequent than in the non-smoker group (39 cases in 30 patients, *P* = 0.017).

### Risk factors for postoperative complications

Based on the univariate analysis, postoperative complications were significantly more commonly found in male patients, smokers, aged ≥ 65 years, and those with an American Society of Anesthesiologists (ASA) score ≥ 3, any comorbidity, preoperative albumin < 35 g/L, complications such as bleeding or pyloric obstruction due to the tumor, total gastrectomy, combined multi-organ resection, operation time ≥ 240 min, intraoperative blood loss ≥ 300 mL, lymph node metastasis, pathological TNM stage III, and perioperative blood transfusion (Table 3). After the multivariate analysis of the abovementioned variables, only smoking (odds ratio [OR] = 1.506, 95% confidence interval [CI] 1.131–2.004, *P* = 0.005), perioperative blood transfusion, operation time ≥ 240 min, combined multi-organ resection, ASA score ≥ 3, and comorbidity were significant predictors for postoperative complications (Table 4). Some other well-known variables, such as the extent of lymphadenectomy and total gastrectomy, were not identified to be independent risk factors in the present study, may be due to the relatively small proportion of patients who underwent < D2 lymphadenectomy and low incidence of postoperative complications. In the internal validation of the predictive model by ROC plot, the AUC value was found to be 0.701 (95% CI 0.662–0.739), indicating that the predictive ability of the present model was good.

**Table 3:** Univariate analysis of possible predictors for postoperative complications following radical gastrectomy for gastric cancer (*n* = 2469)

Variables	Complications (*n* = 225)	Non-complications (*n* = 2244)	*P* value
Sex (male/female)	165/60	1459/785	0.012
Age (years) ≥ 65/< 65	64/161	451/1793	0.003
Body mass index (kg/m^2^) ≥ 25/< 25	41/184	310/1934	0.071
Smoking history; yes/no	119/106	937/1307	0.001
ASA score ≥ 3/< 3	44/181	222/2022	< 0.001
Abdominal surgical history; yes/no	31/194	231/2013	0.106
Comorbidity; yes/no	87/138	650/1594	0.002
Chronic obstructive pulmonary disease	12/213	69/2175	0.070
Preoperative albumin (g/L) ≥35/< 35	151/74	1783/461	< 0.001
Complication due to the tumor ^a^; yes/no	69/156	465/1779	0.001
Operation method; open/laparoscopy	185/40	1719/525	0.056
Extent of gastric resection; subtotal/total	158/67	1730/514	0.021
Extent of lymph node dissection ≥ D2/< D2	213/12	2108/136	0.661
Combined multi-organ resection; yes/no	43/182	177/2067	< 0.001
Operation time (min) ≥ 240/< 240	87/138	447/1797	< 0.001
Intra-operative blood loss (mL) ≥ 300/< 300	59/166	435/1809	0.015
Lymph node metastasis; positive/negative	154/71	1346/898	0.013
pTNM stage ^b^; III/I-II	135/90	1142/1102	0.009
Perioperative blood transfusion; yes/no	96/129	409/1835	< 0.001

*ASA* American Society of Anesthesiologist

^a^Including pyloric obstruction or bleeding

^b^Tumor stages are based on the 8th edition of AJCC TNM classification

**Table 4:** Multivariate analysis of possible predictors for postoperative complications following radical gastrectomy for gastric cancer (*n* = 2469)

Variables	Odds ratio (OR)	95% Confidence interval (CI)	*P* value
Perioperative blood transfusion	2.753	2.038–3.719	< 0.001
Operation time ≥ 240 min	2.037	1.500–2.764	< 0.001
Combined multi-organ resection	1.767	1.184–2.638	0.005
Smoking history	1.506	1.131–2.004	0.005
ASA score ≥ 3	1.687	1.152–2.470	0.007
Any comorbidity	1.362	1.010–1.838	0.043

*ASA* American Society of Anesthesiologist

### Subgroup analysis

Patients in the smoker group were classified into three subgroups depending on the duration of the smoking cessation period prior to surgery. The incidence rate of postoperative complications was also investigated for each group (Table 5). The incidence of overall complications among smokers who ceased smoking ≤ 2 weeks, 2 to 4 weeks, and ≥ 4 weeks preoperatively were 14.0%, 15.0%, and 15.0%, respectively, which were significantly higher than in the non-smoker group (*P* = 0.020). But the incidences were similar among patients with different smoking cessation periods. Pulmonary complications were also more commonly detected in patients who stopped smoking ≤ 2 weeks and 2 to 4 weeks preoperatively, compared with those patients in the non-smoker group or those who had stopped smoking for ≥ 4 weeks. There was no significant difference between the incidence of surgical site infection, bleeding, and other types of complications among each of the subgroups. In addition, when comparing the severity of complications, the ≤ 2 weeks and ≥ 4 weeks subgroups had significantly higher rates of major complications (grade III or greater, *P* = 0.040). Importantly, all of the 5 patients who died within 30 days after surgery in the smoker group were among those who had ceased smoking for ≤ 2 weeks before surgery. Although the differences were not statistically significant, smokers with smoking cessation ≤ 2 weeks appeared to have a possible trend towards a higher risk of perioperative mortality (5/823, 0.6%), compared with non-smokers (3/1413, 0.2%) and smokers with cessation > 2 weeks (0/233) (*P* = 0.131 and 0.233, respectively).

**Table 5:** Postoperative complications based on the length of the smoking cessation period before surgery

Variables	Non-smokers (*n* = 1413)	Smokers (n = 1056)			*P* value
		≤ 2 weeks (*n* = 823)	2 to 4 weeks (*n* = 40)	≥ 4 weeks (*n* = 193)	
Complications^a^	143 (10.1%)	115 (14.0%)	6 (15.0%)	29 (15.0%)	0.020
Surgical site infection	59 (4.2%)	43 (5.2%)	0	14 (7.3%)	0.104
Pulmonary complication	46 (3.3%)	44 (5.3%)	4 (10.0%)	6 (3.1%)	0.019
Bleeding	12 (0.8%)	8 (1.0%)	0	4 (2.1%)	0.385
Others	26 (1.8%)	20 (2.4%)	2 (5.0%)	5 (2.6%)	0.444
Severity of complications^b^					0.040
Minor (grade II)	104 (7.4%)	80 (9.7%)	5 (12.5%)	17 (8.8%)	
Major (grade III or greater)	39 (2.8%)	35 (4.3%)	1 (2.5%)	12 (6.2%)	

^a^Data are number of complications (%), patients may have more than one complications

^b^Based on the Clavien-Dindo severity classification of surgical complications

When considering the impact of cigarette dose on the incidence of complications, smokers were subsequently classified into 3 subgroups (light, moderate, and heavy smokers) according to pack-years at the 25th and 75th percentages: ≤ 20, 20 to 40, and ≥ 40 pack-years (Table 6). Complication rates in the light, moderate, and heavy smokers were 7.9%, 13.8%, and 17.6%, respectively. There was a positive association between the incidence of overall complications and cigarette dose. Moderate to heavy smokers (> 20 pack-years) had a significantly higher risk of suffering from postoperative complications compared with light smokers (≤ 20 pack-years) and non-smokers, as was the incidence of pulmonary problems. Although cigarette dose did not adversely have an impact on the incidence of bleeding and other complications, heavy smokers had a significantly increased risk of surgical site infection. However, the incidence of overall complications, surgical site infection, pulmonary problem, and the severity of complications were all comparable between the light smoker and non-smoker groups. After multivariate analysis, it was shown that the ORs for the incidences of overall complications in light, moderate, and heavy smokers were 0.963, 1.478, and 1.777 (95%CI 0.509–1.820, 1.035–2.111, and 1.227–2.574, respectively; *P* = 0.907, 0.032, and 0.002, respectively), compared with the non-smoker group.

**Table 6:** Postoperative complications based on pack-years of smoking before surgery

Variables	Non-smokers (*n* = 1413)	Smokers (*n* = 1056)			*P* value
		≤ 20 pack-years (*n* = 164)	20 to 40 pack-years (*n* = 522)	≥ 40 pack-years (*n* = 370)	
Complications^a^	143 (10.1%)	13 (7.9%)	72 (13.8%)	65 (17.6%)	< 0.001
Surgical site infection	59 (4.2%)	6 (3.7%)	22 (4.2%)	29 (7.8%)	0.022
Pulmonary complication	46 (3.3%)	2 (1.2%)	31 (5.9%)	21 (5.7%)	0.005
Bleeding	12 (0.8%)	3 (1.8%)	4 (0.8%)	5 (1.4%)	0.521
Others	26 (1.8%)	2 (1.2%)	15 (2.9%)	10 (2.7%)	0.367
Severity of complications^b^					0.001
Minor (grade II)	104 (7.4%)	6 (3.7%)	52 (10.0%)	44 (11.9%)	
Major (grade III or greater)	39 (2.8%)	7 (4.3%)	20 (3.8%)	21 (5.7%)	

^a^Data are number of complications (%), patients may have more than one complications

^b^Based on the Clavien-Dindo severity classification of surgical complications

## Discussion

Although several studies have investigated the influence of smoking and short-term preoperative smoking cessation on postoperative outcomes after various types of surgery, the overall quality of evidence is moderate and limited by the small number of studies contributing to the key analyses, leading to contradictory and perhaps even confusing conclusions [8–16, 20, 25]. The majority of previous studies concluded that smoking adversely affects the short-term outcomes after surgery, and smoking cessation several weeks before surgery was effective in reducing the incidence of complications [8–12, 16]. In contrast, other scholars have argued that short-term smoking cessation before surgery was not associated with a decrease in wound healing and pulmonary or overall postoperative complications [13, 14, 20]. Only one study investigated the influence of preoperative smoking cessation on postoperative complications for GC patients in particular, in which Jung et al. [16] reported that postoperative complications, especially impaired wound healing, pulmonary problems, and leakage, occurred more commonly in smokers than in non-smokers. Further subgroup analysis by this research group found that smoking cessation for at least 2 weeks preoperatively was required to reduce the incidence of complications. However, their conclusions have not been verified by other studies, and the potential impact of cigarette dose has never been investigated in GC patients undergoing curative gastrectomy.

To the best of our knowledge, this is the largest study to date that has evaluated the impact of smoking on postoperative complications and the first study to investigate the attributable risk of cigarette dose on postoperative complications, basing on GC patients undergoing radical gastrectomy. According to previous studies, besides pulmonary problems, impaired wound healing and leakage were also confirmed to be significantly associated with smoking [9, 16]. Tissue hypoxia caused by acute exposure to smoking and lacking proper fibroblast migration to form the healing tissue were considered to be the most important cause of this link [26, 27]. Moreover, heavy smokers had an obviously increased incidence of bleeding requiring transfusion, but the exact reasons for this bleeding remain to be established [10]. Thus, complications were divided into surgical site infection (including wound healing and leakage), pulmonary complication, bleeding, and others in this study, similar with that of the previous study [16]. In this study, we revealed that smoking was an independent risk factor for postoperative complications, especially pulmonary problems. All of the 65 patients developing pneumonia received antibiotic treatments, and 12 among who developed respiratory failure needing ventilation were transferred into the intensive care unit. And finally, 3 patients died due to pneumonia within 30 days after surgery. But they did not differ significantly between the smoker and non-smokers. In addition, severe smoking (≥ 40 pack-years) was identified to be significantly associated with surgical site infections.

The incidences of overall complications were comparable among patients who stopped smoking ≤ 2 weeks, 2 to 4 weeks, and ≥ 4 weeks preoperatively. Compared with the incidence of postoperative pulmonary problems in non-smokers (3.3%), it was comparable with that in those who stopped smoking ≥ 4 weeks before operation (3.1%, *P* = 0.914), but significantly higher in those who stopped smoking < 4 weeks (5.7%, *P* = 0.007). Postoperative pulmonary problems have been identified to be the most costly of all major postoperative complications and resulted in the longest duration of hospital stay, as identified by the National Surgical Quality Improvement Program (NSQIP) [28]. In addition, previous studies have found that patients who underwent upper abdominal surgery were twice as likely to develop pulmonary problems than those undergoing any other surgical category [15]. Given the high incidence of and the significant adverse impact of postoperative pulmonary complications, it is very important for patients who underwent gastrectomy to quit smoking before their operation, which has been identified as a modifiable risk factor. Based on the present study, at least 4 weeks cessation may be needed to prepare for surgery, a finding consistent with the conclusion reported by Lindström et al. [12], who found that smoking cessation 4 weeks before surgery decreased the risk of complications. A meta-analysis of 6 randomized controlled trials and 15 observational studies revealed that each week of preoperative abstinence increased the relative risk reduction of postoperative complications by 19% [22]. And at least 4 weeks of smoking cessation had a significant larger treatment effect than shorter abstinence from smoking. Jung et al. [14] even suggested that as short as 2 weeks of smoking cessation before surgery was effective in reducing its adverse influence on surgical outcomes. One possible explanation is that the acute toxic effect of recent smoke inhalation is a major mechanism for the delayed wound healing and infection risk [8, 16]. Thus, even short-term smoking cessation can reduce the damage caused by acute exposure to smoking and lead to a significant reduction in postoperative complications.

However, other studies have revealed that smoking cessation about half a month, 8 weeks, or even 16 weeks before surgery did not reduce the level of overall complications [13, 14, 20]. In the opinion of these researchers, the chronic cumulative effects of smoking on pulmonary functions, such as increased mucus production and a reduction in immune functions, played a more important role than acute toxic exposure, which could result in postoperative pulmonary infection or even respiratory failure [8]. It has been reported that pulmonary function generally improved after about 8 weeks of smoking cessation [25]. Thus, the optimal duration of smoking cessation prior to an operation to reduce postoperative complications remains poorly defined. Because almost all of the patients underwent surgery within 2 weeks from the diagnosis of GC in our department, the patients with smoking cessation > 2 weeks before operation had stopped smoking when they were admitted, whereas patients with cessation time ≤ 2 weeks were generally current smokers until they were advised to stop smoking during admission. Compared with patients with shorter preoperative smoking cessation period (≤ 2 weeks), those with longer interval (> 2 weeks) seemed to have a higher rate of developing symptoms because of the tumor, such as bleeding and/or vomiting due to pyloric obstruction (26.7% vs 21.1%), although the difference was barely outside the range of significance (*P* = 0.077). Moreover, patients with longer smoking cessation period were significantly older than those with shorter cessation interval (60.17 vs 56.13 years, *P* < 0.001), and with significantly lower preoperative hemoglobin levels (118.54 vs 125.05 g/L, *P =* 0.001), whereas advanced age, lower hemoglobin level, and pyloric obstruction were all well-known adverse predictors for morbidity following gastrectomy [3, 5, 29, 30]. Thus, we hypothesize that although the incidences of postoperative complications were comparable among patients with different smoking cessation period in the present study, the benefit of longer cessation time might be counteracted by the poorer general condition of the patients. Therefore, a prospective randomized large sample size study is needed to confirm this conjecture in the future.

In contrast to smoking status and duration of smoking cessation, the influence of smoking severity on postoperative complications has rarely been investigated. The incidence of postoperative complications was identified to be positively associated with cigarette dose in the present study, as was the surgical site infection. Pulmonary complications were also significantly more common in moderate to heavy smokers (with > 20 pack-years). In a previous study including 20,830 cancer patients to investigate the potential effect of smoking on postoperative outcomes, Gajdos and colleagues [9] divided pack-years into quartiles and split by smoking status. Given the relatively smaller sample size of our study, we classified the smokers into 3 subgroups according to the 25th and 75th cigarette dose. As expected, major adverse events were also significantly more common in heavy smokers, compared with light to moderate smokers or non-smokers. The dose-response relationship was highly suggestive of a causal rather than a mere correlational association. The results were echoed by Hawn et al. [8], who argued that a dose-dependent relationship existed between pulmonary problems and pack-year exposure, and revealed that smoking-associated surgical complications were significantly increased in patients smoking for > 20 pack-years. Similarly, Livingston et al. [31] found that current smoking of > 20 pack-years was related to an increased risk of failure to wean in a series of 575 bariatric patients. In a propensity score-matched analysis of 36,454 patients who underwent plastic surgery, Toyoda et al. [10] concluded that smokers with 11 or more pack-years had significantly higher rates of deep surgical site infections and re-operation following plastic surgery. A dose-dependent association between urine nicotine levels and wound healing impairment in breast reduction patients was also found by Bartsch et al [32] The possible explanation was the chronic cumulative effects of the adverse impact of smoking, such as tissue hypoxia on pulmonary, immune, and wound healing functions [8]. This explanation was indirectly supported by the finding that a high concentration of oxygen following operation decreased the risk of wound healing problems [33]. Therefore, it was reasonable to hypothesize that a high concentration of oxygen during or after gastrectomy for heavy smokers could reduce the incidence of postoperative complications. But further study is needed to unequivocally clarify this suggestion.

The present study has a number of limitations first and foremost being that it was a retrospective and single-institution study. Thus, although smoking was identified to be an independent predictor for postoperative complications, especially pulmonary problems, it does not indicate a causal relationship and further prospective studies are needed. Second, the smoking habits and cigarette dose were only orally obtained by the responsible doctor, instead of through biological monitoring (such as nicotine metabolites), which would have been more objective. Although we recorded the average number of smoked cigarettes and the duration of smoking to calculate the cigarette dose, it should be borne in mind that the content of harmful substances, such as nicotine, in different types of cigarettes is likely to vary significantly, which was not investigated in detail in the present study. Therefore, there is a possibility of misclassification and miscalculation of the risk of complications in the subgroup analysis. Last but by no means the least, the basic clinicopathological characteristics were significantly different in smokers and non-smokers, such as age and gender, which may present as confounders when investigating the relationship between smoking and postoperative complications. A prospective study or retrospective study with large samples using propensity score matching analyses to adjust the potential selecting bias may eliminate this limitation [10].

## Conclusions

The present study has identified that smoking was an independent adverse predictor for postoperative complications, especially for pulmonary problems, in GC patients following radical gastrectomy. It is suggested that at least 4 weeks of smoking cessation prior to surgery is required to decrease postoperative pulmonary problems. There was a positive relationship between the incidence of complications and cigarette dose, and more than 20 pack-years were shown to increase significantly the risk of complications. Therefore, to reduce postoperative complications, both surgeons and patients should pay much attention to preoperative smoking cessation, especially for heavy smokers.

## Abbreviations

ASA: American Society of Anesthesiologists
BMI: Body mass index
CI: Confidence interval
COPD: Chronic obstructive pulmonary disease
FEV1: Forced expiratory volume in 1 s
GC: Gastric cancer
NSQIP: National Surgical Quality Improvement Program
OR: Odds ratio
TNM: Tumor, node, and metastasis
UICC: Union for International Cancer Control
VC: Vital capacity
